# Beneficial Effects of Micronutrient Supplementation in Restoring the Altered Microbiota and Gut–Retina Axis in Patients with Neovascular Age-Related Macular Degeneration—A Randomized Clinical Trial

**DOI:** 10.3390/nu16223971

**Published:** 2024-11-20

**Authors:** Simone Baldi, Giuditta Pagliai, Leandro Di Gloria, Marco Pallecchi, Francesco Barca, Benedetta Pieri, Gianluca Bartolucci, Matteo Ramazzotti, Amedeo Amedei, Gianna Palendri, Francesco Sofi

**Affiliations:** 1 Department of Experimental and Clinical Medicine, University of Florence, 50134 Florence, Italy; simone.baldi@unifi.it (S.B.); giuditta.pagliai@unifi.it (G.P.); 2 Department of Biomedical, Experimental and Clinical Sciences “Mario Serio”, University of Florence, 50134 Florence, Italy; leandro.di.gloria@gmail.com (L.D.G.); matteo.ramazzotti@unifi.it (M.R.); 3 Department of Neuroscience, Psychology, Drug Research and Child Health NEUROFARBA, University of Florence, 50139 Florence, Italy; marco.pallecchi@unifi.it (M.P.); gianluca.bartolucci@unifi.it (G.B.); 4 Complex Operative Unit of Ophthalmology, Palagi Hospital, USL Toscana Centro, 50122 Florence, Italy; francesco.barca@uslcentro.toscana.it (F.B.); benedetta.pieri@uslcentro.toscana.it (B.P.); gpalendri@yahoo.it (G.P.); 5 Network of Immunity in Infection, Malignancy and Autoimmunity (NIIMA), Universal Scientific Education and Research Network (USERN), Florence, Italy; 6 Unit of Clinical Nutrition, Careggi University Hospital, 50134 Florence, Italy

**Keywords:** nAMD, gut microbiota, gut–retina axis, lutein, zeaxanthin, saffron, short-chain fatty acids

## Abstract

**Background/Objectives:** Age-related macular degeneration (AMD) is a leading cause of visual impairment in the elderly and is characterized by a multifactorial etiology. Emerging evidence points to the potential involvement of the gut–retina axis in AMD pathogenesis, prompting exploration into novel therapeutic strategies. This study aims to investigate the effects of some micronutrients (such as lutein and zeaxanthin) and saffron (as a supplement)—known for their anti-inflammatory properties—on ophthalmological and microbial parameters in neovascular AMD (nAMD) patients. **Methods:** Thirty naive nAMD patients were randomized to receive daily micronutrient supplementation alongside anti-VEGF (vascular endothelial growth factor) therapy, or anti-VEGF treatment alone, over a 6-month period, with comparisons made to a healthy control (HC) group (N = 15). Ophthalmological assessments, biochemical measurements, and stool samples were obtained before and after treatment. Gut microbiota (GM) characterization was performed using 16S rRNA sequencing, while short-chain fatty acids (SCFAs), medium-chain fatty acids (MCFAs), and long-chain fatty acids (LCFAs) were analyzed with a gas chromatography–mass spectrometry protocol. **Results:** Compared to HC, nAMD patients exhibited reduced GM alpha diversity, altered taxonomic composition, and decreased total SCFA levels, in addition to elevated levels of proinflammatory octanoic and nonanoic acids. Micronutrient supplementation was associated with improved visual acuity relative to the group treated with anti-VEGF alone, along with a decrease in the total amount of MCFAs, which are metabolites known to have adverse ocular effects. **Conclusions:** In conclusion, despite certain limitations—such as the limited sample size and the low taxonomic resolution of 16S rRNA sequencing—this study highlights compositional and functional imbalances in the GM of nAMD patients and demonstrates that micronutrient supplementation may help restore the gut–retina axis. These findings suggest the therapeutic potential of micronutrients in enhancing ocular outcomes for nAMD patients, underscoring the complex interaction between GM and ocular health.

## 1. Introduction

Age-related macular degeneration (AMD) is the leading cause of visual impairment in the over-65-year-old population of industrialized countries, affecting approximately 170 million people globally [1,2,3]. This complex, multifactorial disease involves genetic and environmental factors [4,5]. AMD presents primarily in two forms, both of which can result in central vision loss and blindness due to the degeneration of photoreceptor cells [6]. The first, referred to as dry AMD, is characterized by the accumulation of extracellular deposits (such as lipids, vitronectin, inflammatory, or amyloid proteins) between Bruch’s membrane and the retinal pigment epithelium, leading to drusen formation. These drusen—small yellow or white spots on the retina—may progress to retinal atrophy or, in around 20% of cases, to the second AMD form: wet or neovascular AMD (nAMD) [7]. nAMD is characterized by the abnormal growth of blood vessels breaching Bruch’s membrane. This condition is driven by various mechanisms, including the impact of oxidized low-density lipoproteins (LDL), which can contribute to deposit buildup within the retinal pigment epithelium (RPE). Oxidized LDL impairs the RPE’s capacity to degrade photoreceptor outer segments (OS) by slowing phagosome maturation, leading to inefficient OS breakdown. Consequently, undigested lipids and proteins accumulate within the RPE, promoting the formation of toxic compounds, such as A2E, that can damage RPE cells [7].

Current treatments for nAMD focus on inhibiting the abnormal blood vessel growth associated with choroidal neovascularization (CNV). Early treatments like laser photocoagulation aimed to limit CNV progression but often caused some degree of permanent vision loss due to unintended retinal damage. To date, anti-VEGF (vascular endothelial growth factor) therapies, which work by binding to and neutralizing VEGF, have become the standard treatment. These therapies offer significant visual improvement for many patients and effectively halt disease progression in the majority. [8]

Moreover, recent research has identified immune system alterations, oxidative stress, and obesity as crucial contributors to AMD pathogenesis [9,10]. To date, numerous studies have highlighted the potential benefits of dietary supplementation with micronutrients that possess antioxidant and anti-inflammatory properties for reducing the risk of AMD development [11,12,13]. Notably, the AREDS2 study definitively demonstrated the efficacy of dietary supplementation with lutein and zeaxanthin in reducing the risk of progression of early-stage AMD [14]. The administration of lutein and zeaxanthin, which absorb blue light and neutralize free radicals and reactive oxygen species in the macula, has been associated with increased macular pigment optical density (MPOD), improved visual acuity, and a reduced risk of retinal ageing [15]. Moreover, other micronutrients, such as vitamins E and C, can help prevent the progression of maculopathy by offering protection against oxidative stress and supporting GM homeostasis [16,17], while oral zinc supplementation can reduce complement-mediated inflammation in the retinal pigment epithelium, a key factor in AMD etiology [18]. Additionally, saffron (*Crocus sativus*), containing active components such as crocin, safranal, crocetin, and picrocrocin, has demonstrated antioxidant and anti-inflammatory effects, leading to significant improvements in the retinal function of AMD patients [19,20,21]. Considering that the retina is an extension of the brain both anatomically and developmentally, the hypothesis of a gut–retina axis has recently emerged, drawing parallels with the extensively studied bidirectional communication between the gut and the brain [22,23]. More in detail, in AMD, the “gut-retina axis” plays a critical role through several mechanisms linking GM alterations to retinal health. Changes in the GM can increase intestinal permeability, allowing metabolites and microbial products to enter the bloodstream and affect retinal immune cells, promoting inflammation within the retina [24]. For example, inflammatory responses to lipopolysaccharides (LPS) have been shown to accelerate retinal degeneration [25]. Additionally, specific bacterial species—such as *Anaerotruncus*, *Oscillibacter*, and *Ruminococcus torques*—were elevated in AMD patients and associated with glutamate degradation and increased arginine biosynthesis pathways. This is significant, as both altered glutamate levels and increased arginine are linked to retinal dysfunction and degeneration [26].

Hence, in this study, the GM composition and function in nAMD patients were evaluated in comparison to healthy subjects. Additionally, given the dual potential of micronutrients to exert effects through direct antioxidant mechanisms and GM modulation, the impact of a novel micronutrient supplementation containing lutein, zeaxanthin, and saffron (as a supplement) on ophthalmological parameters and microbial features in naïve nAMD patients was investigated.

## 2. Materials and Methods

### 2.1. Patients

A total of N = 30 naïve nAMD patients (19 F:11 M; mean age 77.8 years) and N = 15 healthy controls (HC) (8 F:7 M; mean age 75.2 years) were enrolled in accordance with the following exclusion criteria: use of antibiotics or continued use of pre- or probiotics in the 2 months before enrollment; use of other treatments (medications or nutritional programs) that affect body weight, food intake, and/or energy expenditure; and diagnosis of any ocular disease.

Additionally, at baseline, participants’ lifestyle factors were assessed through specific questionnaires covering eating habits, physical activity, bowel health, pharmacological treatments, and smoking or alcohol consumption.

In this three-arm randomized, controlled trial, with one arm comprising healthy subjects, eligible participants were randomly divided into two groups. Fifteen nAMD patients were allocated to the intervention group and received, over 6 months, intravitreal injections of anti-VEGF (Aflibercept 2 mg, 0.05 mL) at a fixed regimen, along with a daily administration of a micronutrient mix containing lutein (10 mg), zeaxanthin (2 mg), vitamin C (80 mg), vitamin E (12 mg), zinc (10 mg), and saffron (20 mg) as a supplement. The other fifteen patients were assigned to the active comparator group, receiving only the intravitreal anti-VEGF treatment at a fixed regimen for 6 months. For both groups, an ophthalmological examination with the best correct visual acuity (BCVA), biomicroscopy, and swept optical coherence tomography (OCT) were performed at enrollment and after 6 months. Additionally, blood samples for interleukin (IL)-6, IL-10, and tumor necrosis factor-α (TNF-α) quantification, as well as stool samples for gut microbiota (GM) compositional and functional analysis, were collected at baseline for both nAMD groups and after six months for the intervention group. Stool samples were immediately stored at −80 °C until analysis.

The study research adhered to the principles of the Declaration of Helsinki, and informed consent was obtained from all enrolled patients. Study procedures were approved by the Ethics Committee of the Tuscany Region, Careggi University Hospital (n.16281_bio of 16 February 2021) and were registered at clinicaltrials.gov (identifier: NCT06391411).

### 2.2. Fecal Microbiota Characterization

Genomic DNA was extracted using the DNeasy PowerSoil Pro Kit (Qiagen, Hilden, Germany) from frozen (−80 °C) stool samples, according to the manufacturer’s instructions. The quality and quantity of the extracted DNA were assessed using both NanoDrop ND-1000 (Thermo Fisher Scientific, Waltham, MA, USA) and Qubit Fluorometer (Thermo Fisher Scientific, Waltham, MA, USA), and then it was frozen at −20 °C. Next, total DNA samples were sent to IGA Technology Services (Udine, Italy), where amplicons of the variable V3–V4 region of the bacterial 16S rRNA gene, obtained through primers 341F and 805R, were sequenced in paired-end mode (2 × 300 cycles) on the Illumina MiSeq platform, according to the Illumina 16S Metagenomic Sequencing Library Preparation protocol.

Demultiplexed sequence reads were processed using QIIME2 2022.8 [27].

Demultiplexed sequence reads were processed using QIIME2 (version 2022.8). The Cutadapt tool was employed to remove sequencing primers and any reads without primers, while DADA2 [28] was used for filtering, merging paired-end reads, and removing chimeras, with low-quality nucleotides trimmed from both forward and reverse reads. Additional checks for cross-amplified host DNA were conducted with Bowtie2 v.2.2.5. Hence, ASVs (amplicon sequence variants) were generated, and taxonomic assignments were performed through the Scikit-learn multinomial Naive Bayes classifier re-trained on the SILVA database (release 138) V3–V4 hypervariable region.

Finally, every ASV associated with genera with an average relative abundance below a 0.005% cutoff were excluded to reduce sequencing contaminants and improve statistical reliability [29,30]. Further details about the data analysis are available at https://github.com/LeandroD94/Papers/tree/main/2024_ADM_microbiota_gut_retina_axis, accessed on 1 March 2024.

### 2.3. Fecal Short-, Medium-, and Long-Chain Fatty Acids Evaluation by GC-MS Analysis

The qualitative and quantitative evaluation of fecal short (SCFAs)-, medium (MCFAs)-, and long-chain fatty acids (LCFAs) was performed through our previously described method [31].

Briefly, just before the analysis, stool samples were thawed and mixed with a 0.25 mM sodium bicarbonate solution (1:1 *w/v*) in a 1.5 mL centrifuge tube. Then, the resulting suspensions were sonicated for 5 min and centrifuged at 5000× *g* for 10 min, and then the supernatants were collected. The SCFAs, MCFAs, and LCFAs were finally extracted as follows: an aliquot of 100 µL of sample solution (corresponding to 0.1 mg of stool sample) was added to 50 µL of internal standards mixture, 1 mL of tert-butyl methyl ether, and 50 µL of HCl 6 M + 0.5 M NaCl solution in a 1.5 mL centrifuge tube. Subsequently, each tube was shaken in a vortex apparatus for 2 min and centrifuged at 10,000× *g* for 5 min, and lastly, the solvent layer was transferred to an autosampler vial and processed three times.

### 2.4. Statistical Analysis

Statistical analyses of bacterial communities were performed in R 4.2.2 using the packages phyloseq 1.40.0, vegan 2.6-2, DESeq2 1.36.0, and other packages satisfying their dependencies. The packages ggplot2 3.3.6, ggh4x 0.2.2, and ggpubr 0.40 were used to plot data and results. ASV saturation analysis was conducted on each sample using the function rarecurve (step 100 reads), with further processing to identify saturated samples (arbitrarily defined as saturated samples with a final slope in the rarefaction curve, with an increment in ASV number per reads <1 × 10^−5^. The observed richness and Shannon indices were used to estimate the bacterial alpha diversity in each sample using the function estimate_richness from phyloseq. Pielou’s evenness index was calculated using the formula E = S/log(R), where S is the Shannon diversity index, and R is the observed ASV richness in the sample. Differences in alpha diversity indices were analyzed using the Mann–Whitney test. Principal coordinate analysis (PCoA) was performed using the Hellinger distance on Hellinger-transformed genera abundances. PERMANOVA and Betadisper analyses assessed the statistical significance of the beta diversity distances and dispersions. At different taxonomic ranks, the differential analysis of abundances was computed with DESeq2 on raw count data, and *p*-values (adjusted through the Benjamini–Hochberg method) lower than 0.05 were considered statistically significant. Taxa with a DESeq2 baseMean value <50 were excluded from the results to reduce noise. Furthermore, GraphPad Prism (v.8) was used for the statistical analysis of fecal SCFA, MCFA, and LCFA abundances between nAMD patients and either HC or patients without micronutrient supplementation using the Mann–Whitney test (*p*-values < 0.05 considered significant).

Further details about the data analysis are available at https://github.com/LeandroD94/Papers/tree/main/2024_ADM_microbiota_gut_retina_axis, accessed on 1 March 2024.

Statistical analyses of microbial metabolites and biochemical parameters were performed in GraphPad Prism, with data reported as mean ± standard deviation (SD) or percentage and interquartile range, as appropriate. The Mann–Whitney test compared intervention and control groups, and a general linear model for repeated measures was applied to evaluate the treatment effects. Statistical significance was defined as *p* < 0.05.

## 3. Results

### 3.1. Gut Microbiota Characterization

**Figure 1:**
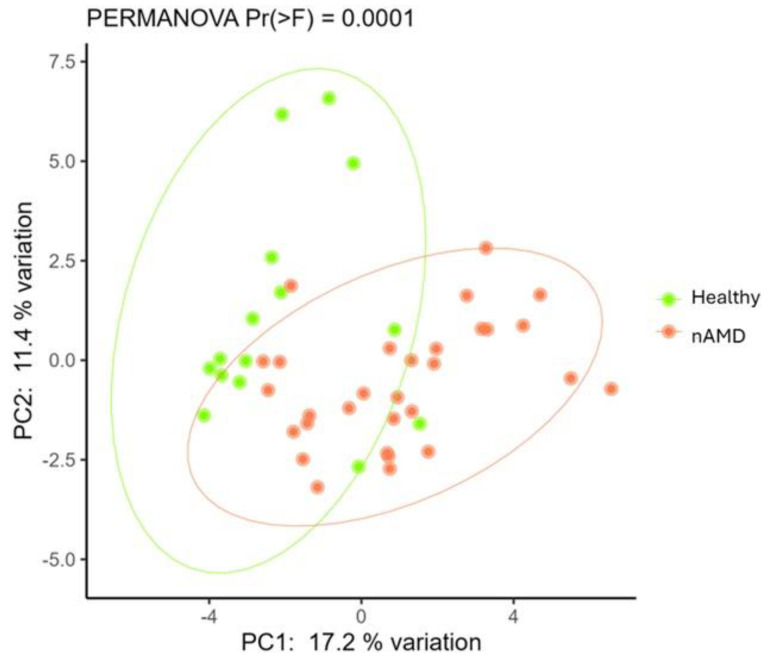
Principal coordinate analysis (PCoA) computed using Hellinger distance on transformed genera abundances of stool samples among HC and nAMD patients.

First, an assessment was conducted to determine whether patients with nAMD exhibited a different intestinal microbiota structure compared to HC. The PCoA plot, computed using the Hellinger distance metric, showed a significant separation (PERMANOVA, *p* < 0.0001) among stool samples from HC and nAMD patients (Figure 1).

**Figure 2:**
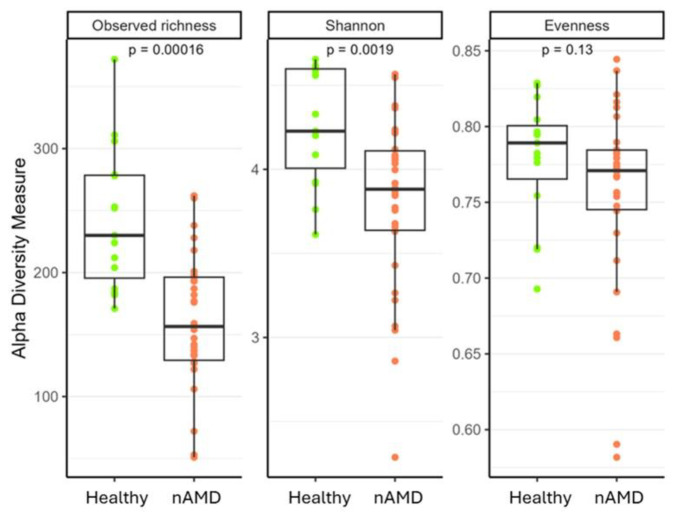
Box plots showing fecal alpha diversity indices (observed ASV richness, Shannon index, and Pielou’s evenness) between HC and nAMD patients.

Notably, statistically significant beta diversities were observed at all taxonomic ranks (Table S1). Additionally, as displayed in Figure 2, nAMD patients demonstrated a significantly decreased intestinal microbial alpha diversity (observed ASV richness, *p* = 1.6 × 10^−4^ Shannon index, *p* = 0.001), compared to HC.

**Figure 3:**
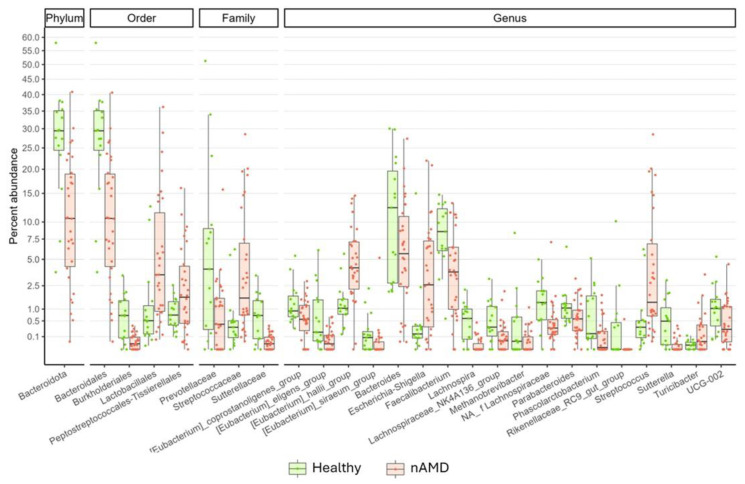
Boxplot displaying the results of differential abundances analysis between HC and nAMD patients. The Y-axis, reporting the percent abundance of each taxon, is scaled to improve the readability of lower abundances. All results have an adjusted. *p* < 0.05.

Subsequently, differential abundance analyses were performed at all taxonomic ranks, identifying several taxa with differential abundances in stool samples from HC and nAMD patients (Figure 3 and Table S2).

In detail, compared to HC, nAMD patients reported reduced fecal abundances of members belonging to the Bacteroidota phylum; Bacteroidales and Burkholderiales orders; Prevotellaceae and Sutterellaceae families; and *Eubacterium_coprostanoligenes_group*, *Eubacterium_eligens_group*, *Eubacterium_siraeum_group*, *Bacteroides*, *Faecalibacterium*, *Lachnospira*, *Lachnospiraceae_NK4A136_group*, *Methanobrevibacter*, an unidentified genus of the Lachnospiraceae family, *Parabacteroides*, *Phascolarctobacterium*, *Rikenellaceae_RC9_gut_group*, *Sutterella*, and UCG-002 genera. Conversely, nAMD patients exhibited increased levels of bacteria belonging to the *Lactobacillales* and *Peptostreptococcales-Tissierellales* orders; *Streptococcaceae* family; and *[Eubacterium]_hallii_group*, *Escherichia-Shigella*, *Streptococcus*, and *Turicibacter* genera.

### 3.2. Fecal SCFAs, MCFAs, and LCFAs Profiles

**Figure 4:**
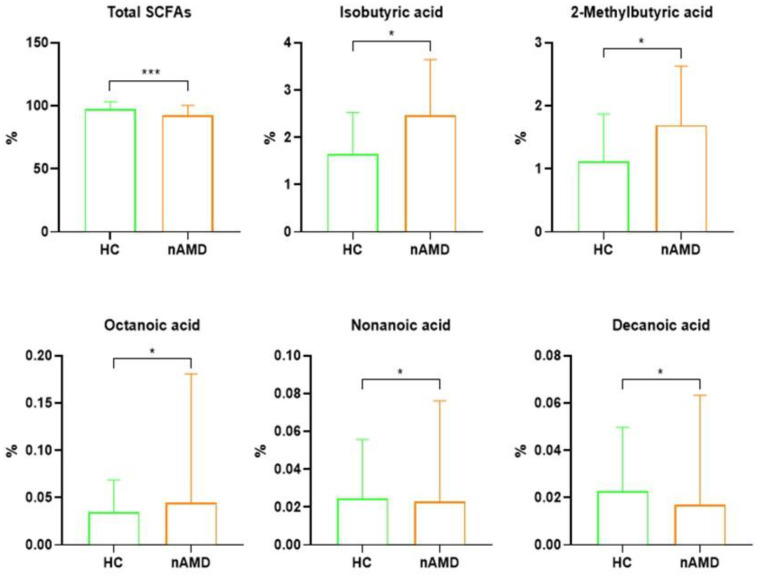
Bar plots representing the significant fecal acid abundances among HC and nAMD patients. Analyses were assessed using the Mann–Whitney test, and *p*-values less than 0.05 were considered statistically significant. The asterisks (*) represent *p*-values; * *p* < 0.05, *** *p* < 0.001.

Using a GC-MS approach, the abundances of microbial-derived SCFAs (acetic, propionic, butyric, isobutyric, isovaleric 2-methylbutyric, and valeric acids), MCFAs (hexanoic, isohexanoic, heptanoic, octanoic, nonanoic, decanoic, dodecanoic acids), and LCFAs (tetradecanoic, hexadecanoic, and octadecanoic acids) were assessed in stool samples from both HC and nAMD patients. To account for potential variations due to the total amount of each metabolite, the comparisons were conducted on the percentage acids’ compositions (Table S3). As depicted in Figure 4, in addition to a significant reduction in the total SCFA levels (*p* = 1 × 10^−4^), nAMD patients had increased levels of isobutyric (*p* = 0.025), 2-methylbutyric (*p* = 0.027), octanoic (*p* = 0.034), and nonanoic (*p* = 0.043) acids but reduced abundances of decanoic acid (*p* = 0.001), compared to HC.

### 3.3. Micronutrient Supplementation

Given the documented GM compositional and functional dysbiosis in nAMD patients, potentially reflecting an altered gut–retina axis, the impact of a six-month administration of a micronutrient mix containing lutein, zeaxanthin, and saffron (as a supplement) on microbial features, as well as biochemical, inflammatory, and ophthalmological parameters, was investigated. Baseline characteristics of the enrolled nAMD patients, randomized into either the intervention or control group, are detailed in Table S4. No significant differences were observed between the two study groups at baseline.

### 3.4. Impact of the Intervention on Biochemical Parameters

Although information for nAMD patients in the control group was not available, micronutrient administration did not lead to significant changes in the biochemical parameters, including the evaluated cytokines (Table S5).

### 3.5. Intervention Effects on Ophthalmological Parameters

**Figure 5:**
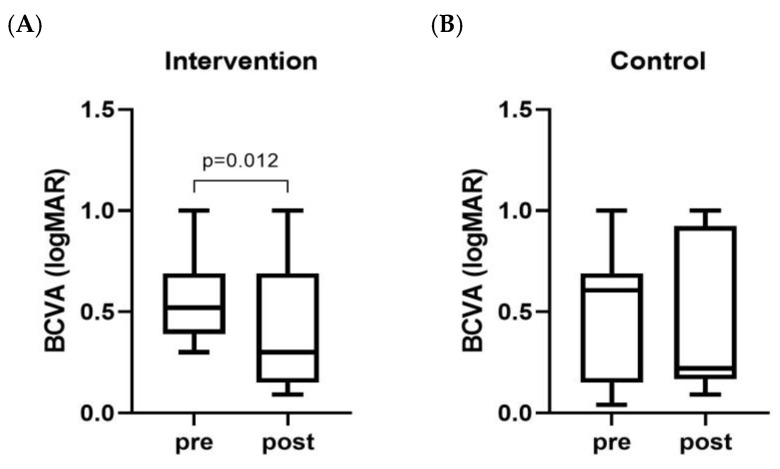
Visual acuity measurements of nAMD patients assigned to intervention (**A**) or control (**B**) groups.

Concerning ophthalmological parameters, the effects on BCVA, expressed as the median (IQR) of the log of the minimum angle of resolution (logMAR), were evaluated. Notably, micronutrient supplementation led to a significant improvement in visual acuity (BCVA pre: 0.52 (0.39–0.60), BCVA post: 0.30 (0.15–0.69); *p* = 0.012) (Figure 5A). In contrast, no significant changes were observed in the control group (BCVA pre: 0.60 (0.15–0.69), BCVA post: 0.69 (0.15–1.00); *p* = 0.882) (Figure 5B).

**Figure 6:**
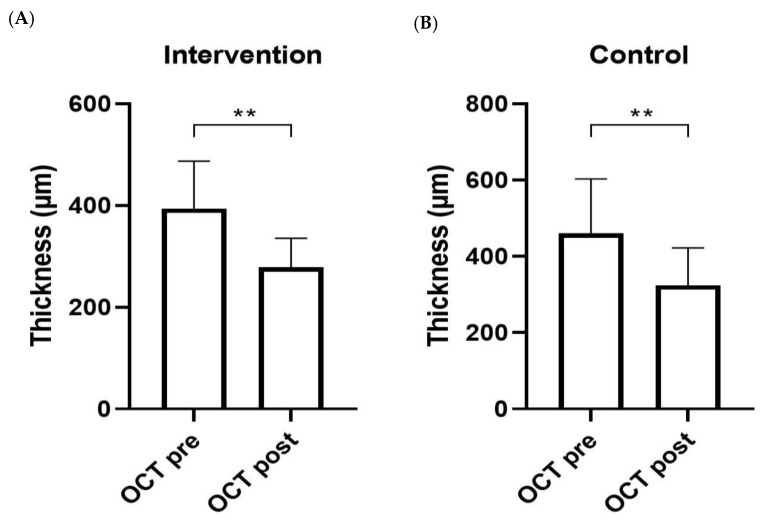
OCT of nAMD patients assigned to intervention (**A**) and control (**B**) groups. Analyses were assessed using the Mann–Whitney test, and *p*-values less than 0.05 were considered statistically significant. The asterisks (*) represent *p*-values; ** *p* < 0.01.

Additionally, a significant reduction in OCT was observed in both the intervention (*p* = 0.001) (Figure 6A) and control (*p* = 0.001) (Figure 6B) groups.

### 3.6. Intervention Effects on Fecal Microbiota

**Figure 7:**
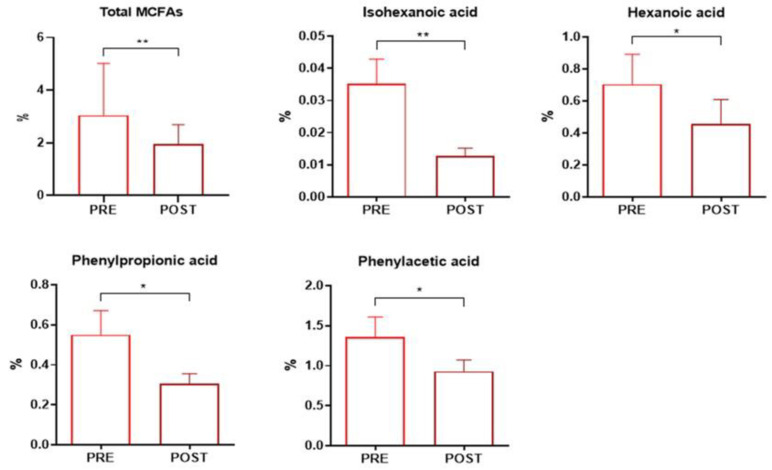
Bar plots representing the significant fecal acid abundances in nAMD patients pre- and post-micronutrient supplementation. Analyses were assessed using the Mann–Whitney test, and *p*-values less than 0.05 were considered statistically significant. The asterisks (*) represent *p*-values; * *p* < 0.05, ** *p* < 0.01.

As shown in Figure S1A, no significant differences were observed in the various alpha diversity indices following micronutrient supplementation, and no distinct separation was observed between pre- and post-intervention samples (PERMANOVA, *p* = 0.7505) (Figure S1B). However, in terms of GM function, micronutrient supplementation led to a significant reduction in the total amount of MCFAs (*p* = 0.008), as well as in levels of isohexanoic (*p* = 0.005), hexanoic (*p* = 0.037), phenylacetic (*p* = 0.040), and phenylpropionic (*p* = 0.018) acids (Figure 7 and Table S6).

## 4. Discussion

Vision loss represents a significant global disability, with AMD identified as the leading cause of irreversible blindness in industrialized countries [32]. Environmental factors, including diet and lifestyle, have been recognized as major contributors to the pathobiology of AMD, exhibiting a strong association between the risk and progression of AMD and factors such as high-fat diet, increased body mass index, and elevated waist/hip ratio [9,33]. Moreover, various studies have documented the disruption of the blood–retinal barrier during the development or progression of chronic retinal diseases, including diabetic retinopathy, retinitis pigmentosa, and AMD [22]. Consequently, the impairment of the intestinal barrier resulting from GM dysbiosis, along with an increased inflammatory response associated with eye diseases, can lead to the excessive translocation of gut-derived microbes and metabolites (particularly SCFAs) into the bloodstream, which may then reach the intraocular environment [34,35].

In this scenario, the existence of bidirectional communication between the gut and the retina has recently come under the spotlight.

Therefore, despite certain limitations—including the limited number of enrolled patients and the low taxonomic resolution of 16S rRNA sequencing— this study aimed to enhance the understanding of the microbiota–gut–retina axis by characterizing the GM composition and function in naïve nAMD patients and investigating the impact of new micronutrient supplementation on the compositional and functional features of the GM.

Consistent with the findings of Zhang and colleagues, the β-diversity analysis demonstrated distinct differences in gut bacterial compositions between the two groups, accompanied by significantly reduced intestinal alpha diversity in patients with nAMD [36].

Moreover, several bacterial taxa exhibited differential abundance between the two groups. Specifically, nAMD patients showed significantly reduced proportions of Bacteroidota, Bacteroidales, and Prevotellaceae members. Notably, while Bacteroidota is recognized as one of the top five abundant phyla in the gut, Zhang et al. reported increased levels in AMD patients, which contrasts with the current results [36]. Additionally, bacteria belonging to the Bacteroidales class, known for their protective role against AMD, were enriched in low-glycemic diet-fed mice [37].

At the genus level, decreased abundances of Eubacterium_coprostanoligenes_group, Eubacterium_eligens_group, Eubacterium_siraeum_group, Bacteroides, Faecalibacterium, Methanobrevibacter, Rikenellaceae_RC9_gut_group, and Sutterella were documented in nAMD patients, compared to HC.

In healthy people, *Eubacterium coprostanoligenes* has been linked to the regulation of cholesterol metabolism, contributing to reduced serum cholesterol levels [38]. Meanwhile, *Eubacterium eligens* and *Eubacterium siraeum* have been associated with lower insulin secretion [39] and increased HDL-cholesterol production [40], respectively, further supporting the hypothesized relationship between AMD and obesity.

In line with these findings, other studies have reported reduced abundances of *Bacteroides* spp., *Faecalibacterium* spp., and Rikenellaceae members in AMD patients, compared to healthy subjects [24,41]. In addition, nAMD patients exhibited a reduced abundance of SCFA-producing genera, such as *Lachnospira*, *Lachnospiraceae NK4A136 group*, *Parabacteroides*, and *Phascolarctobacterium*, all recognized for their beneficial effects on the host [42,43,44].

Increased abundances of Lactobacillales, Peptostreptococcales-Tissierellales, Streptococcaceae, *Eubacterium_hallii_group* spp., *Escherichia-Shigella* spp., *Turicibacter* spp., and *Streptococcus* spp. were observed in nAMD patients, compared to HC. Supporting these findings, previous studies have reported the enrichment of *Lactobacillus* and *Escherichia-Shigella* genera in AMD patients [33,38], while increased abundances of *Streptococcus* spp. and *Eubacterium hallii* have been associated with various eye diseases and obesity [45,46].

In addition to compositional dysbiosis, a functional intestinal alteration was documented in nAMD patients, compared to HC, characterized by a significant reduction in SCFA production and an increase in pro-inflammatory octanoic and nonanoic acids. SCFAs contribute to host health through several mechanisms, including the maintenance of intestinal barrier integrity, mucus production, and histone deacetylase (HDAC) inhibition and modulation of inflammation [47]. Recent findings have also demonstrated their role in reducing both extra- and intra-ocular inflammation [35,48].

Considering these documented compositional and functional gut imbalances, a novel micronutrient administration based on lutein, zeaxanthin, and saffron (as a supplement) was administered to the enrolled nAMD patients to evaluate its potential impact on their ophthalmological and microbial features.

Lutein and zeaxanthin are the only dietary carotenoids that accumulate in the retina, specifically in the macula.

Their known protective effects are primarily associated with defending against oxidative stress and scavenging free radicals, acting as potent biological antioxidants.

They also serve as efficient blue-light filters, quenching reactive oxygen species (ROS) formed during photoexcitation [49]. Additionally, zeaxanthin may play a role in the inflammatory response, contributing to the treatment or prevention of diseases like allergies. Furthermore, it exhibits anticancer, anti-osteoporotic, and ophthalmologic effects. These properties are mediated through various cellular and molecular mechanisms, including the activation or inhibition of cell receptors, modulation of signaling pathways, and effects on gene expression [50].

Of note, supportive therapies based on lutein and zeaxanthin supplementation have demonstrated beneficial effects in delaying the progression of eye diseases, including nAMD [51]. Furthermore, other studies have shown promising effects of saffron in slowing AMD progression, attributed not only to its important antioxidant and anti-inflammatory properties but also to its capacity to modulate metalloproteinase expression and reduce extracellular matrix disorganization [52].

Consistently, the six-month micronutrient administration resulted in improved visual acuity, compared to the control group. In line with these findings, a 2-year randomized, double-blinded, placebo-controlled trial conducted by Huang and colleagues demonstrated that long-term lutein supplementation increased MPOD and visual sensitivities in early AMD patients [53]. Furthermore, recent meta-analyses have highlighted that lutein/zeaxanthin supplementation for longer than 1 year led to significantly greater improvements in visual acuity, compared to the placebo, showing a dose-response relationship. [51,54]. Interestingly, the micronutrient supplementation in this study resulted in beneficial optical improvements within a shorter intervention duration than currently published studies, likely due to the additional effects of saffron.

Saffron is known for its potent antioxidant and anti-inflammatory properties, primarily due to its rich content of carotenoids, such as crocin and crocetin, as well as safranal. These compounds help neutralize free radicals, reducing oxidative stress and protecting cells from damage that can contribute to aging, cancer, and other diseases. Additionally, crocin and safranal are thought to inhibit key inflammatory pathways, including the activity of enzymes like cyclooxygenase (COX), which play a central role in inflammation [55].

Accordingly, a 3-month randomized, double-blind, placebo-controlled trial by Riazi and colleagues documented that, although no changes in macular thickness were found, significant increases in visual acuity and contrast sensitivity were observed in the saffron-treated group, compared to the control group [56].

Regarding the GM, despite no significant taxonomic differences, it was noted that micronutrient supplementation did not result in changes in SCFA abundances. To date, no studies have explored the impact of carotenoid-based micronutrient supplementations on GM composition in AMD patients. Only a recent study conducted by Dai and colleagues tested the effects of β-carotene, lutein, lycopene, and astaxanthin on intestinal microflora using an in vitro fermentation model, documenting a significant enhancement in total SCFA production alongside increases in *Roseburia* spp. and *Parasutterella* spp., as well as a decrease in *Collinsella* spp. [57].

Interestingly, this study found that micronutrient supplementation led to a significant beneficial reduction in the total amount of MCFAs, including isohexanoic, hexanoic, phenylacetic, and phenylpropionic acids. Although various anaerobic bacteria possess essential enzymes for their de novo synthesis, MCFAs are generally derived from the diet, particularly from milk and dairy products, and act as important regulators of energy metabolism, membrane trafficking, and gene expression [58,59]. MCFAs are also potential ligands for free fatty acid receptor 1 (FFAR1), also known as G-protein-coupled receptor 40 (GPR40), a receptor associated with proinflammatory functions, documented in both in vitro and in vivo studies [60].

Of note, FFAR1 is expressed in the retina, and Heckel and colleagues demonstrated that high levels of MCFAs detected by FFAR1 in photoreceptors could suppress the activity of transcription factor EB activity, a master regulator of autophagy and lipid metabolism, leading to the increased production of VEGFA, which drives compensatory yet pathological angiogenesis [61].

## 5. Conclusions

In conclusion, this study demonstrates the beneficial effects of micronutrient supplementation in nAMD patients, such as enhanced visual acuity and reductions in the abundance of pro-inflammatory medium-chain fatty acids (MCFAs). Although further studies are warranted to more thoroughly define the relationship between the gut microbiota and retinal health, these findings emphasize the complex interplay between the gut microbiome and ocular health, providing valuable insights into potential innovative interventions for AMD management.

## Data Availability

The data presented in this study are deposited in the NCBI Gene Expression Omnibus (GEO) repository, accession number GSE261732. The clinical trial was registered at clinicaltrials.gov; identifier: NCT06391411.

## Supplementary Materials

The following supporting information can be downloaded at: https://www.mdpi.com/article/10.3390/nu16223971/s1. Figure S1. Box plots reporting alpha diversity indices (observed ASV richness, Shannon index, and Pielou’s evenness) pre- and post-prebiotic administration (A). Principal coordinates analysis (PCoA) conducted with the Hellinger distance on pre- and post-prebiotic samples (B). Lines link paired samples, and statistical differences were assessed using the Wilcoxon signed-rank test. Table S1. PERMANOVA tests at all taxonomic ranks between stool samples of HC and nAMD patients. Table S2. Significant differentially abundant taxa in stool samples of nAMD patients, compared to HC. The table reports the Log2FoldChange and adjusted *p*-values less than 0.05. Table S3. Fecal SCFA, MCFA, and LCFA abundances of HC and nAMD patients. Comparisons were assessed with the Mann–Whitney test, and *p*-values less than 0.05 were considered statistically significant. Table S4. Baseline characteristics of the enrolled nAMD patients according to the randomization. Table S5. Effects of the micronutrient supplementation on biochemical parameters. Table S6. Fecal SCFA, MCFA, and LCFA abundances of nAMD patients pre- and post-prebiotic administration. Comparisons were assessed with the paired Wilcoxon test, and *p*-values less than 0.05 were considered statistically significant.
