# Supplementary material for: Beneficial Effects of Micronutrient Supplementation in Restoring the Altered Microbiota and Gut–Retina Axis in Patients with Neovascular Age-Related Macular Degeneration—A Randomized Clinical Trial
Source: Nutrients. 2024 Nov 20;16(22):3971. doi: 10.3390/nu16223971 (PMC11597754; doi:10.3390/nu16223971)
Supplement: Supplementary file 1 [file nutrients-16-03971-s001.zip › nutrients-3291824-supplementary.pdf]

SUPPLEMENTAL MATERIAL

SUPPLEMENTARY FIGURES

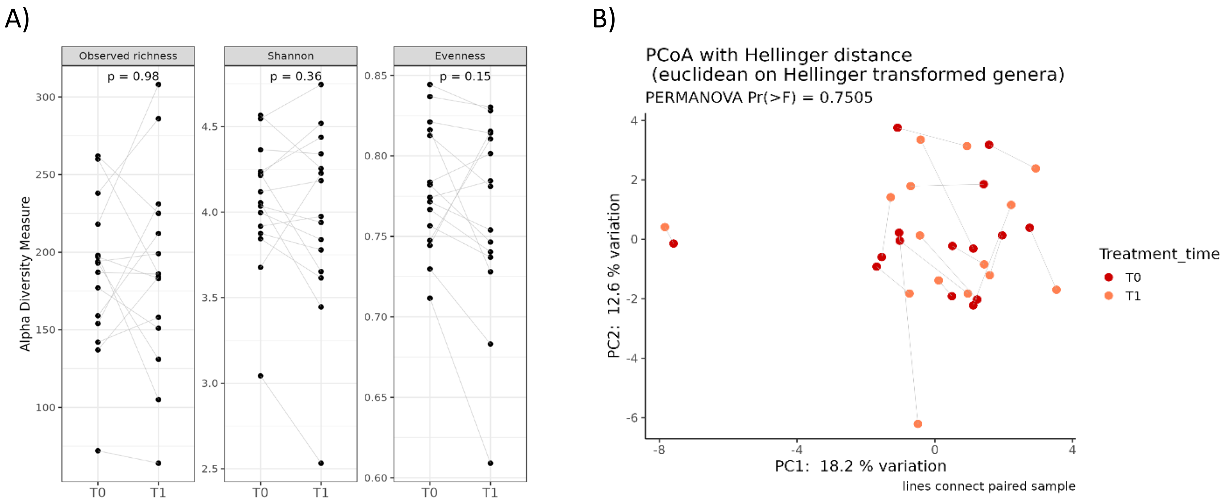

Figure S1. Box plots reporting alpha diversity indices (Observed ASV richness, Shannon index, Pielou's evenness) pre- and post- prebiotic administration (A). Principal coordinates analysis (PCoA) conducted with the Hellinger distance on pre- and post- prebiotic samples (B). Lines link paired samples and statistical differences were assessed using the Wilcoxon signed-rank test.

SUPPLEMENTARY TABLES

Table S1. PERMANOVA tests at all taxonomic ranks between stool samples of HC and nAMD patients.

Rank	SumsOfSqs	MeanSqs	F.Model	R2	P value
Phyla	50.76238	50.76238	6.26935	0.127246	< 0.0005
Classes	90.22976	90.22977	4.89976	0.102292	< 0.0002
Orders	56.98939	56.98940	4.97812	0.103758	< 0.0013
Families	86.45961	86.45961	3.51754	0.075617	< 0.0004
Genera	145.0469	145.0469	3.73349	0.081069	< 0.0001

Table S2. Significant differentially abundant taxa in stool samples of nAMD patients compared to HC. The table report the Log2FoldChange and adjusted p-values less than 0.05.

Log2FoldChange	padj	Regulation		
1.3022	0.0120	*down*	Bacteroidota	**Phylum**
1.7663	0.0009	*down*	Bacteroidales	

4.5039	$7.73e^{-7}$	*down*	Burkholderiales	**Order**
-2.5671	0.0015	*up*	Lactobacillales	
-1,7659	0.0319	*up*	Peptostreptococcales-Tissierellales	
3.5630	0.0149	*down*	Prevotellaceae	**Family**
-3.3068	0.0008	*up*	Streptococcaceae	
4.2335	$1.14e^{-5}$	*down*	Sutterellaceae	
4.3182	0.0184	*down*	*[Eubacterium]_coprostanoligenes_group*	**Genus**
4.1094	0.0023	*down*	*[Eubacterium]_eligens_group*	
-1.3549	0.0391	*up*	*[Eubacterium]_hallii_group*	
4.3182	0.0184	*down*	*[Eubacterium]_siraeum_group*	
1.6627	0.0084	*down*	*Bacteroides*	
-2.9867	0.0013	*up*	*Escherichia-Shigella*	
1.9381	0.0078	*down*	*Faecalibacterium*	
4.8367	0.0061	*down*	*Lachnospira*	
2.7135	0.0406	*down*	*Lachnospiraceae_NK4A136_group*	
3.7762	0.0497	*down*	*Methanobrevibacter*	
2.8881	0.0001	*down*	*NA_ f Lachnospiraceae*	
2.2600	0.0005	*down*	*Parabacteroides*	
3.7975	0.0168	*down*	*Phascolarctobacterium*	
28.8819	$1.13e^{-29}$	*down*	*Rikenellaceae_RC9_gut_group*	
-3.9003	$9.74e^{-6}$	*up*	*Streptococcus*	
5.8011	0.0001	*down*	*Sutterella*	
-3.5845	0.0197	*up*	*Turicibacter*	
2.2356	0.0187	*down*	*UCG-002*	

Table S3. Faecal SCFAs, MCFAs and LCFAs abundances of HC and nAMD patients. Comparisons were assessed with the Mann-Whitney test and p-values less than 0.05 were considered statistically significant.

**Percentage of each faecal SCFA, MCFA and LCFA** (mean (IQR); %)	**nAMD**	**HC**	**p value**
Total SCFAs	92.85 (3.82)	97.35 (2.12)	0.0001
Acetic acid	59.98 (10.34)	63.68 (7.48)	0.1046
Propionic acid	16.33 (5.74)	15.26 (5.28)	0,9949
Butyric acid	8.61 (4.24)	11.63 (4.24)	0,0808
isoButyric acid	2.46 (1.81)	1.65 (1.20)	0,0251
isoValeric acid	1.80 (1.53)	1.23 (1.05)	0,0542
2-MethylButyric acid	1.70 (1.37)	1.12 (0.83)	0,0277
Valeric acid	2.39 (1.58)	2.28 (1.07)	0,5394
Total MCFAs	2.82 (1.89)	2.70 (1.32)	0,1351
Isohexanoic acid	0.04 (0.04)	0.03 (0.02)	0,7400
Hexanoic acid	0.60 (0.87)	0.73 (1.10)	0,4413
Heptanoic	0.14 (0.24)	0.11 (0.20)	0,9569
Octanoic	0.04 (0.02)	0.03 (0.02)	0,0344
Nonanoic	0.02 (0.01)	0.02 (0.02)	0,0435
Decanoic	0.02 (0.00)	0.02 (0.02)	0,0017
Dodecanoic	0.05 (0.01)	0.03 (0.01)	0,1843
Phenylacetic	1.25 (0.78)	1.25 (0.63)	0,2903
Phenylpropionic	0.70 (0.34)	0.47 (0.35)	0,6724
Total LCFAs	3.91 (3.10)	3.90 (3.01)	0.7437
Tetradecanoic	0.09 (0.11)	0.09 (0.04)	0,8516
Hexadecanoic	1.90 (1.68)	1.58 (1.21)	0,5553
Octadecanoic	2.17 (2.23)	3.91 (3.10)	0.9904

Table S4. Baseline characteristics of the enrolled nAMD patients according to the randomization.

Variable	All	VEGF therapy	VEGF therapy +micronutrients	p-value
Age, y	77.83 ± 8.45	78.40 ± 9.27	77.26 ± 7.82	0.601
Female sex, n (%)	19 (63.3)	9 (60)	10 (66.6)	0.999
White blood cells, ×109/L	6.68 ± 1.53	6.54 ± 1.50	6.81 ± 1.61	0.568
Red blood cells, ×1012/L	4.68 ± 0.55	4.83 ± 0.62	4.54 ± 0.44	0.525
Hemoglobin, g/dL	13.71 ± 1.50	13.58 ± 1.54	13.82 ± 1.51	0.674
Platelets, ×106/L	256.34 ± 72.32	245.92 ± 78.05	266.06 ± 67.78	0.133
Glucose, mg/dL	109.96 ± 21.97	113.07 ± 23.45	107.06 ± 20.89	0.204
HDL-cholesterol, mg/dL	67.79 ± 15.45	64.14 ± 15.17	71.20 ± 15.43	0.255
LDL-cholesterol, mg/dL	124.86 ± 34.42	122.21 ± 34.01	127.33±35.80	0.771
Triglycerides, mg/dL	101.96 ± 30.91	108.28 ± 38.17	96.6 ± 21.92	0.524
IL-6	3.71 ± 1.93	3.44 ± 1.65	3.96 ± 2.19	0.530
IL-10	3.59 ± 2.54	3.15 ± 0.18	4.01 ± 3.53	0.960
TNF-α	24.62 ± 36.58	15.62 ± 0.10	33.02 ± 50.20	0.150

Table S5. Effects of the micronutrient supplementation on biochemical parameters.

Variable	Intervention pre	Intervention post	p-value
White blood cells, ×109/L	6.81 ± 1.61	6.37 ± 1.30	0.625
Red blood cells, ×1012/L	4.61 ± 0.37	4.55 ± 0.39	0.094
Hemoglobin, g/dL	14.05 ± 1.26	13.50 ± 1.48	0.161
Platelets, ×106/L	266.06 ± 67.78	265.14 ± 90.68	0.529
Glucose, mg/dL	107.06 ± 20.89	101.78 ± 10.87	0.635
HDL-cholesterol, mg/dL	68.40 ± 23.04	69.35 ± 14.60	0.250
LDL-cholesterol, mg/dL	127.33 ± 35.80	128.21 ± 30.09	0.760
Triglycerides, mg/dL	96.06 ± 21.92	102.28 ± 40.19	0.541

IL-6	3.96 ± 2.19	3.00 ± 0.00	0.500
IL-10	4.01 ± 3.53	3.86 ± 2.85	0.999
TNF-α	33.02 ± 50.20	22.62 ± 13.47	0.937

Table S6. Faecal SCFAs, MCFAs and LCFAs abundances of nAMD patients pre- and post- prebiotic administration. Comparisons were assessed with the paired Wilcoxon test and p-values less than 0.05 were considered statistically significant.

**Percentage of each faecal SCFA, MCFA and LCFA** (mean (IQR); %)	**nAMD pre**	**nAMD post**	**p value**
Total SCFAs	92.57 (3.92)	89.67 (8.04)	0.168
Acetic acid	56.02 (16.84)	59.85 (17.78)	0.445
Propionic acid	17.59 (5.34)	14.32 (5.99)	0.073
Butyric acid	8.61 (4.24)	6.58 (7.98)	0.187
isoButyric acid	3.11 (1.08)	2.85 (1.41)	0.073
isoValeric acid	2.32 (1.27)	1.97 (1.29)	0.168
2-MethylButyric acid	2.22 (1.30)	1.90 (1.11)	0.207
Valeric acid	2.67 (1.60)	2.21 (1.17)	0.120
Total MCFAs	3.05 (1.91)	1.96 (1.16)	0.008
Isohexanoic acid	0.04 (0.04)	0.01 (0.01)	0.005
Hexanoic acid	0.69 (0.96)	0.46 (0.66)	0.037
Heptanoic	0.14 (0.24)	0.09 (0.18)	0.090

Octanoic	0.03 (0.04)	0.02 (0.02)	0.311
Nonanoic	0.04 (0.01)	0.02 (0.02)	0.750
Decanoic	0.02 (0.01)	0.01 (0.01)	0.931
Dodecanoic	0.03 (0.01)	0.03 (0.03)	0.220
Phenylacetic	1.36 (0.61)	0.93 (0.86)	0.040
Phenylpropionic	0.55 (0.52)	0.31 (0.16)	0.018
Total LCFAs	4.38 (3.84)	8.37 (7.41)	0.055
Tetradecanoic	0.09 (0.08)	0.18 (0.25)	0.261
Hexadecanoic	1.95 (1.67)	3.17 (3.24)	0.083
Octadecanoic	2.21 (1.67)	5.01 (5.25)	0.063
